# New Mixed Methods Approach for Monitoring Community Perceptions of Ebola and Response Efforts in the Democratic Republic of the Congo

**DOI:** 10.9745/GHSP-D-21-00144

**Published:** 2021-06-30

**Authors:** Giulia Earle-Richardson, Eva Erlach, Vivienne Walz, Ombretta Baggio, Molly Kurnit, Cheick Abdoulaye Camara, Christina Craig, Lucia Robles Dios, Daiva Yee, Gnakub Norbert Soke, Ialijaona Voahary, Christine E. Prue

**Affiliations:** a Centers for Disease Control and Prevention, Atlanta, GA, USA.; b International Federation of the Red Cross and Red Crescent Societies, Geneva, Switzerland.

## Abstract

**Background:**

Efforts to contain the spread of Ebola in the eastern Democratic Republic of the Congo (DRC) during the 2018–2020 epidemic faced challenges in gaining community trust and participation. This affected implementation of community alerts, early isolation, contact tracing, vaccination, and safe and dignified burials. To quickly understand community perspectives and improve community engagement, collaborators from the DRC Red Cross, the International Federation of the Red Cross, and the U.S. Centers for Disease Control and Prevention explored a new method of collecting, coding, and quickly analyzing community feedback.

**Methods:**

Over 800 DRC Red Cross local volunteers recorded unstructured, free-text questions and comments from community members during community Ebola awareness activities. Comments were coded and analyzed using a text-coding system developed by the collaborators. Coded comments were then aggregated and qualitatively grouped into major themes, and time trends were examined.

**Results:**

Communities reported a lack of information about the outbreak and the response, as well as concerns about the Ebola vaccination program and health care quality. Some doubted that Ebola was real. The response used the feedback to revise some community engagement approaches. For example, 2 procedural changes that were followed by drops in negative community responses were: using transparent body bags, which allayed fears that bodies or organs were being stolen, and widening the eligibility criteria for Ebola vaccination, which addressed concerns that selectively vaccinating individuals within Ebola-affected communities was unfair.

**Discussion:**

This system is unique in that unstructured feedback collected by local volunteers in the course of their work was rapidly coded, analyzed, and given to health authorities for use in making course corrections throughout the response. It provides a platform for local voices to be heard throughout an emergency response and provides a mechanism for assessing the effects of program adjustments on community sentiments.

## Abstract (precis)

The Red Cross community feedback system enabled rapid collection and analysis of extensive verbal feedback during an Ebola outbreak in eastern DRC. Using this information, Ebola response leaders adapted strategies to address community concerns. In an epidemic, community feedback is critical to ensure that response strategies are accepted and appropriate.

## Teaser

**Key Findings:**

- Ebola-affected communities in the eastern Democratic Republic of the Congo had questions about the outbreak, doubts about the reality of Ebola, and concerns about health care and the Ebola vaccination program.
- In peak outbreak areas, beliefs that Ebola response teams were stealing organs and bodies declined after burial teams introduced transparent body bags. Similarly, calls for making vaccination “more fair” declined after Ebola vaccination eligibility was expanded.

**Key Implications:**

- The model for recording community feedback provided rapid, ongoing comments from neighborhoods and villages where Ebola response activities were underway, allowing people to share perceptions, questions, and concerns in their own words. Local volunteers were central to the model's success because they were involved in the rapid collection, coding, and interpretation of feedback and applying it to response activities.
- Collecting continuous community feedback throughout an emergency response enables response teams to answer questions, consider suggestions, and adapt interventions to better meet community needs and preferences. Local health departments everywhere could employ this approach to enhance community engagement.

Résumé en français à la fin de l'article.

## INTRODUCTION

By June 21, 2020, the Democratic Republic of Congo (DRC) had reported 3,470 probable or confirmed cases of Ebola virus disease (EVD) since the beginning of the outbreak in 2018. Of these, 2,287 persons died and 1,171 recovered.^1^ On July 30, 2018, the DRC Ministry of Health led a coalition of international agencies, governments, and nongovernmental organizations in a coordinated Ebola response, despite a uniquely challenging context of political conflict and violence.^2^ The response focused on 4 main interventions to stop EVD spread: (1) identifying possible cases and isolating, testing, and treating the patients quickly at EVD treatment centers; (2) finding, quarantining, and monitoring people who had exposure to an EVD case for 21 days (as well as people having had contact with those contacts); (3) offering vaccination to contacts and their contacts; and (4) conducting safe and dignified burials* in outbreak-affected communities.

As part of this response, local Red Cross volunteers in Ebola-affected communities undertook risk communication and community engagement activities (e.g., community meetings, “phone-in” radio shows, mobile “cinema” presentations) and made home visits in selected communities. During these activities, volunteers recorded any comments from community members about Ebola, such as questions, beliefs, observations, rumors, or suggestions for improving the government-led response to the epidemic.

### Prior Efforts With Community Engagement and Community Feedback in Epidemic Response

Although the concepts are not new in humanitarian settings, community engagement has been slower to be fully incorporated into epidemic response structures,^3^^,^^4^ despite growing evidence that community engagement strengthens epidemic control.^5^^–^^9^ The hesitation to embrace community engagement in epidemic emergency response may stem from the fact that authentic engagement requires an investment of time and resources in understanding community needs, as well as a willingness to change epidemic control strategies based on community feedback.

Understanding community needs through some form of community listening (e.g., surveys, focus groups, interviews, rapid ethnography) must be done in a very compressed timeframe and often in extremely challenging field conditions. Even when it is accomplished, engagement only succeeds if social scientists can get actionable information into the hands of decision makers who are willing to be advised in their work. Ideally, community engagement in emergency response is supported by a regular “feedback loop,” in which community reactions to epidemic control activities are continuously monitored and addressed.^10^^,^^11^

In the context of an epidemic, understanding community needs must be done in a very compressed timeframe and often in extremely challenging field conditions.

### Context of the North Kivu, DRC Ebola Outbreak

The Ebola epidemic that began in North Kivu province of DRC in 2018 was described by the country's minister of health as “the longest, most complex and deadliest” in the country's history.^12^ The urban location of the early cases contributed to its persistence, as did the social and political turmoil in the region. According to the World Health Organization (WHO), there were 420 attacks on health facilities in eastern DRC during the outbreak period,^13^ and security reviews identified 140 armed groups active in the area.^14^ Kidnappings and killings of civilians were also frequently reported.^15^^,^^16^ As a result, for much of the North Kivu Ebola response, WHO-led operations were located hundreds of miles from where cases were occurring.^17^ Mistrust of the national government is widespread, as the North Kivu region has long been a stronghold of political opposition. This mistrust intensified in December 2018, when the government excluded several areas in North Kivu from voting in national elections due to the Ebola outbreak.^18^

### The Rapid Community Feedback Collaborators

While gathering community feedback was not a new idea for IFRC as it looked to support the Red Cross of DRC in Ebola control, it needed a way to systematically and rapidly gather feedback to apply qualitative analytic methods to produce reports that could offer specific, actionable recommendations. The CDC Ebola Response Social and Behavioral Science task force had recently created a similar rapid assessment system to support the Zika emergency response in Puerto Rico and the U.S. Virgin Islands in 2016.^11^^,^^19^ The Red Cross of DRC was a trusted local partner, which had been promoting health and assisting with health emergencies and epidemics for many years. In August 2018, the DRC Red Cross, IFRC, and CDC collaborated to create a method for entering free-text notes into a Microsoft Excel spreadsheet and coding them to allow for rapid aggregation, analysis, and reporting. The current analysis identifies the major themes of community concern during the 2018–2020 Ebola outbreak in DRC and describes how the information was used by the Red Cross and Ebola response leaders to better address community concerns.

## METHODS

### Recruitment and Training of Red Cross Volunteers

At the outset of the Ebola outbreak in North Kivu, IFRC and local health authorities selected neighborhoods near where individuals with Ebola virus disease had been identified and where community engagement approaches needed to be intensified to prevent and control the spread of the virus. Red Cross volunteers were activated if already present in the targeted communities and/or new ones recruited where activities needed to be urgently scaled up. New volunteer training and refresher trainings included the following topics: Ebola facts, principles of community engagement and accountability, listening skills, and how to accurately and ethically collect and record community feedback.^20^ In addition to consulting community and neighborhood leaders before approaching homes or initiating public awareness activities, Red Cross team leads were trained to maintain an awareness of the local situation and to have a low threshold for ending activities on a given day if tensions became apparent.^†^

### Community Feedback Collection During Community Engagement Activities

During the outbreak period, the Red Cross had more than 800 volunteers working across 29 health zones to raise awareness about Ebola. While the number of volunteers participating in Ebola awareness-raising varied widely by health zone and by month (according to the severity of the Ebola outbreak at the time and security conditions), typically volunteers worked in groups of 8 to 10 (in teams of 2), with 1 team lead in a given neighborhood or rural village, supported by a field supervisor, who covered 4 or 5 teams. Three days per week, volunteer teams met with field supervisors to review key Ebola messages and to receive their assigned areas for home visits. Each team visited 15 homes and held an informal conversation about Ebola, sharing basic facts about the disease, treatment, and prevention and answering any questions. While one volunteer led the conversation, the other took notes, writing down anything anyone said related to Ebola that was a question; a statement of rumor, experience, or belief; a suggestion; or an expression of appreciation. The information noted was recorded without any direct questioning of the community members. In addition, volunteers organized public presentations followed by informal discussions. Feedback from these events was also recorded. Conversations were conducted in local languages (primarily Swahili and Kinandé), and comments were written in the local language or French. Volunteers explained to participants that comments were being written down so that the volunteers and the Ebola response could understand their questions, comments, and suggestions.

During the outbreak period, the Red Cross had more than 800 volunteers working across 29 health zones to raise awareness about Ebola.

### Feedback Collection Form

Red Cross volunteers wrote all community comments on a collection form divided into 5 sections: statements (including rumors, beliefs, and observations), questions, suggestions, expressions of appreciation, and other (Photo 1). To manage the large volume of comments coming at the same time, volunteers were permitted to annotate comments with a multiplier (e.g., 2, 3), when a single comment was repeated 1 or more times by different people. Volunteers gave their completed forms to their field supervisor, who collated them and gave them to data managers for entry and coding in an Excel spreadsheet.

### Coding Scheme and Process

Within the existing categories of comment type (statements, questions, suggestions, expressions of appreciation, and other), text codes were created to distinguish and describe the meaning of comments. The development of the coding scheme was a 3-way effort over several months among Red Cross field volunteers, who had the best insight into the local cultural and political context; IFRC (international and local staff), who had the most experience with using community feedback data for decision making; and CDC staff, who had the technical expertise on qualitative coding system design and analysis. (See Supplement for a complete list of text codes.) Once the scheme was finalized, coding was performed using a bilingual Excel spreadsheet containing preprogrammed drop-down coding menus. CDC scientists coded the feedback in the first months of the collaboration and then trained Red Cross volunteers and IRFC staff to code. From that time forward, the first round of coding was performed by Red Cross volunteers and IFRC staff in DRC in French, and then all codes were reviewed by CDC coders, with 20% being reviewed by senior CDC coders. Discrepant codes and potential adjustments to the coding scheme were discussed and resolved on weekly French-language conference calls.

**Figure:**
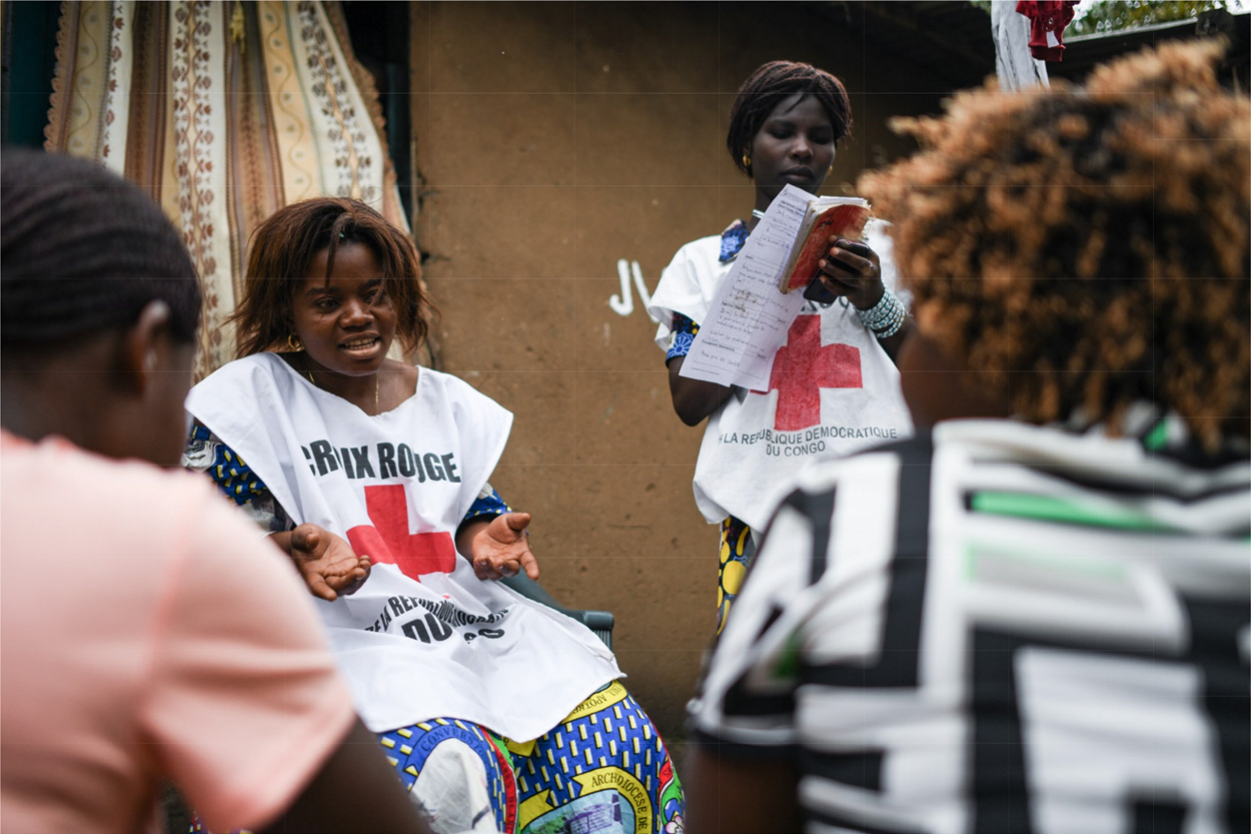
Red Cross volunteers in the Democratic Republic of the Congo talking to families in Bunia about Ebola and gathering information about their questions and concerns. These types of interactions will help improve how humanitarian actors, including Red Cross, address community concerns. © 2021 Corrie Butler/International Federation of Red Cross

### Data Analysis During the Response

Initially, CDC generated simple data reports that aggregated codings by comment type (statement, question, suggestion, etc.) and category. Using Excel functions and macros, these reports were generated very quickly; CDC was able to produce more than 80 rapid data reports for use by Red Cross and response leaders. Later on, collaborators identified specific topical areas for more in-depth analysis, resulting in 23 “deep dive” reports and numerous other reports in which CDC applied additional Excel features (such as the Vlookup function and text search macros) for special analyses. IFRC and Red Cross leaders championed the sharing and use of these data for informing, reviewing, and revising field activities of the response task forces (Photo 2). The coded community feedback was also shared with all response partners through an interactive dashboard created by IFRC. Results from community feedback analyses were frequently triangulated with results from studies by the Ebola response social science team.^5^ Since community feedback was collected continuously across the entire outbreak region, it was a good complement to these more structured and geographically limited studies collected at a single point in time.^21^

Results from community feedback analyses were frequently triangulated with results from more structured and geographically limited studies and complemented their findings.

### Analysis Methods for This Report

Analysts reviewed comments for the period August 2018 through February 2020 in health zones in which there had been an Ebola case in the previous 30 days. Codes were qualitatively grouped into themes according to their meaning, with independent review by 2 other analysts. Themes were then rank-ordered by the total number of coded comments within each theme. In a second, exploratory analysis, frequencies of comments relating to Ebola response activities in 4 health zones (Beni, Mabalako, Katwa, and Butembo) were graphed over time, along with weekly EVD case counts. If marked changes in comment frequencies around Ebola response topics were identified, Ebola intervention program history was reviewed to see if the pattern could be explained by changes made in response activities.

**Figure:**
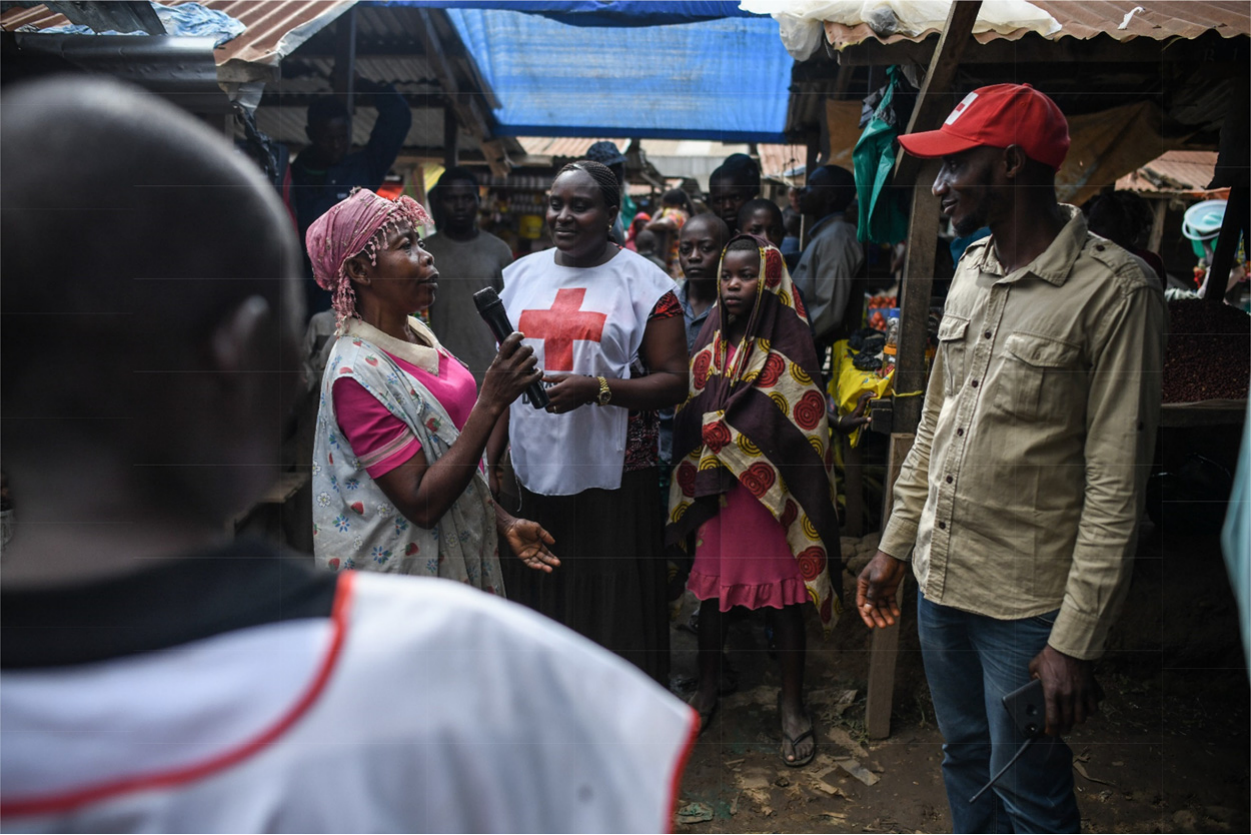
Red Cross volunteers in the Democratic Republic of the Congo provide a sensitization campaign in the biggest market in Bunia. This is a regular activity with communities, providing critical information about Ebola and how to prevent other major diseases. © 2020 Corrie Butler/International Federation of Red Cross

### Ethical Considerations

The Red Cross/IFRC community engagement work was considered evaluation, not research by the DRC Ebola response. This determination is consistent with U.S. definitions.^22^ All work adhered to the International Federation of the Red Cross and Red Crescent Societies' code of conduct and ethical treatment of community members policy.^23^^,^^24^

## RESULTS

**FIGURE 1:**
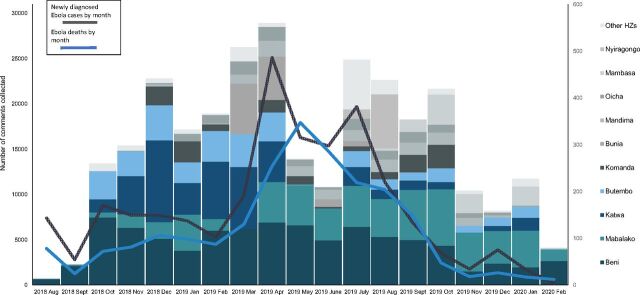
Community Feedback Comments in Ebola-Affected Health Zones^a^ by Health Zone, Month, and Monthly Ebola Case Counts and Deaths,^b^ Eastern Democratic Republic of the Congo, August 2018 to February 2020 (N=292,232) Abbreviations: HZ, health zone. ^a^Health zones include Goma, Rwampara, Kalunguta, Masereka, Musienene, and Kayna. ^b^Ebola deaths may be cases that were diagnosed in previous months. Thus, in some months the number of deaths may be greater than the number of cases.

During the review period, there were 292,232 comments collected from communities experiencing Ebola cases within the previous 30 days. Since some comments were coded with more than 1 text code (e.g., if a comment addressed 2 issues), the total set of coded comments was larger (n=315,820). Figure 1 shows the number of comments collected from 16 Ebola-affected health zones over the epidemic period. The majority of comments (78%) came from 4 health zones: Beni, Mabalako, Katwa, and Butembo.

Just over one-third of coded comments were statements (including rumors, beliefs, and observations, 35% of all comments), with slightly fewer questions (29%) and suggestions (27%). The remaining comments (9%) were nearly all expressions of appreciation. As shown in Table 1, the 5 most common themes were (1) Ebola epidemic and Ebola reality—requests for updates about the progress of the epidemic, and doubts about its reality in one's community; (2) medical diagnosis and treatment—questions and concerns about Ebola treatment centers and Ebola treatment and health care costs and quality; (3) Ebola vaccine and vaccination—perceived unfairness of the “ring” vaccination strategy (for ring vaccination definition, see WHO's Ebola Vaccine Frequently Asked Questions^25^), belief in a “good” and a “bad” vaccine, and doubts about vaccine safety; (4) Ebola response feedback—both positive (appreciation) and negative (doubt and mistrust) comments about the response implementation and response staff; and 5) Ebola profit and politics—perceived personal profit and political motives behind the Ebola response.

**TABLE.** Community Feedback Comments in Ebola-Affected Health Zones by Theme and Code, Eastern DRC, August 2018 to February 2020 (N=315,820)

Themes, Codes (Type^a^)	N	%	Representative Quotations
**1: Ebola epidemic, EVD reality**	**56,672**	**18**	
Questions about the Ebola epidemic (Q)	13,110		*Since the beginning of the response to EVD how many confirmed cases of Ebola have there been? When will Ebola end?* *Why does your Ebola pick…mothers and children?* *Does Ebola really exist here in [my community]?*
Perceptions, beliefs about Ebola outbreak locally (RBO)	9,485		*EBOLA doesn't exist here in [my community].* *The Ebola you're talking about here is not the Ebola we saw on the television of Equateur because we never saw someone who has blood on his body.*
Ebola does not exist (RBO)	6,574		*Ebola doesn't exist. Ebola is not real, it's to postpone the elections.* *It's politics, it's not real.*
**2: Medical diagnosis, treatment**	**47,175**	**15**	
Questions about EVD diagnosis, treatment (Q)	11,071		*Are there any medicines that can cure the Ebola virus disease?* *After how many days can a sick person who goes to the ETC be healed?* *Why would they not want to construct the ETC here among us at [my community]?*
Improve health care (S)	10,747		*We need to bring in real doctors like the doctors who worked at the beginning of the outbreak, since there were recoveries then.* *Free care should continue until the end of this epidemic*
Mistrust of Ebola treatment center (RBO)	7,694		*The health care workers are currently killing people and taking their bodies.* *A person can arrive at the hospital in good health, but in a few minutes he dies.*
**3: Ebola vaccine, vaccination**	**36,093**	**11**	
Expand or modify vaccination program (S)	14,106		*Vaccinate the whole population against Ebola disease.* *We call on the government and the WHO to vaccinate us without discrimination.* *Set up vaccination centers in all neighborhoods.*
Vaccine suspicions (RBO)	7,149		*There are two types of vaccine: injection of Ebola virus disease and injection of vaccine for recovery.* *Your vaccine causes abortion in pregnant women.* *The Ebola vaccine will kill us in 5 years.*
Questions about unfair vaccine distribution (Q)	6,388		*Why aren't you vaccinating the entire population?* *Are there not means to vaccinate all the social categories of the DRC?*
**4: Ebola response feedback**	**33,319**	**11**	
Questions about response processes (Q)	11,152		*Why make so much money available for vehicle hire, luxury hotels, etc. [rather] than multiplying the machines or laboratories that analyze or test for Ebola?* *How come others raise awareness and bring us handwashing units, yet you, you come with empty hands?* *Why are the hospitals being guarded by armed men?*
Thank you, not further specified (A)	6,996		*We thank the response team for their work in the field.* *The community thanks the response team for the service provided; thanks to it the community has been saved.*
Statements about response staff (RBO)	6,498		*We trust the rescuers, they convinced us that the disease exists.* *We doubt the disease today since the agents of response do not want to proclaim the end of the Ebola virus disease…* *People on the outbreak response have their personal interests.*
**5: Ebola profit and politics**	**30,606**	**10**	
Ebola is a “business” (or someone is profiting) (RBO)	12,771		*Ebola can't end because the agents' lives have changed; they earn money from it.* *We want the responders to be replaced because those who are there now earn a lot and don't want Ebola to disappear for good.*
Ebola is a political tool (RBO)	9,652		*Ebola is a conspiracy of whites in collaboration with our politicians.* *We know that the Ebola disease exists, but it is politicized.*
Ebola is used for harming people (by political leaders or through political action) (RBO)	5,131		*The response teams are not there to save the lives of the population, but rather for their private interest and to kill the Nande people.* *Ebola is politics of the Congolese government; our authorities want to exterminate us by spreading Ebola.*
**Grand total**	**315,820**	**100**	

Abbreviations: DRC, Democratic Republic of the Congo; ETC, EVD treatment center; EVD, Ebola virus disease; WHO, World Health Organization.

^a^ Comment text code type: Q, question; RBO, statement of rumor, belief, or observation; S, suggestion; A, appreciation.

Two individual codes that were not part of the 5 major themes stood out because they accounted for more than 10,000 comments each: “Thank you for Ebola awareness efforts” (n=16,475) and “Provide handwashing station(s)” (n=15,218).

### Changes in Leading Comment Codes Over Time Within Health Zones

**FIGURE 2:**
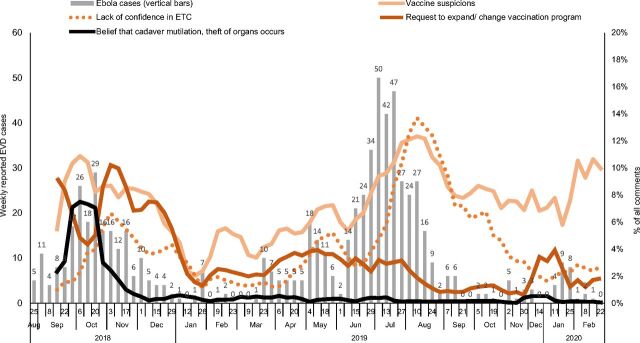
Frequencies^a^ of Comments Related to Ebola Response Activities,^b^ Beni Health Zone, September 2018 to February 2020^c^ Abbreviations: ETC, Ebola testing center; EVD, Ebola virus disease. ^a^Frequencies are expressed as 4-week rolling averages of the percentage of all weekly comments within each code. ^b^Three additional codes—*recommendation to improve health care*, *comments about responders*, and *questions about burials*—are not shown because they did not show any appreciable change over the period. Another code, *contact tracing*, received too few comments overall to show over time. ^c^There were 2 time segments: November 16–29, 2019, and December 21, 2019 to January 4, 2020, in which no data were collected.

In all 4 zones, the percentages of comments about response strategies fluctuated over time. As shown in Figure 2 (Beni health zone), increases in the frequency of comments about different response strategies occurred during or just after an increase in reported EVD cases. This pattern was generally present in Mabalako, Katwa, and Butembo health zones (not shown) as well, although these 3 health zones had notably lower proportions of comments about response efforts overall.

As shown in Figure 2, comments about beliefs in mutilation or theft of organs or cadavers declined over time, and suggestions to expand vaccination to more people had a more subtle drop. These 2 patterns were repeated in the other 3 health zones, except for Mabalako, which experienced a spike in comments suggesting expanded vaccination that coincided with a sudden spike in EVD cases in May 2019. Notable changes did not occur in comment frequencies related to Ebola treatment centers or those related to contact tracing. As this an exploratory scan for patterns that might be associated with changes in Ebola response activities, we are limited in what we can conclude about those activities based on the absence of a pattern.”

### How Collected Data Were Used for Decision Making

Red Cross leaders used weekly data summaries to facilitate discussions and reinforce volunteer Ebola knowledge. In addition, the IFRC presented regular summaries to local Ebola response commissions, who were particularly interested in understanding leading types of misinformation and concerns about response activities. Reports for the highest levels of the response were frequently combined with other analyses and had particular importance in being one of the few sources to provide verbatim comments.

At each of these levels, community feedback led to relatively rapid changes in practice. Red Cross volunteers began using an “answers to frequently asked questions” document,^26^ shared through the Red Cross DRC WhatsApp chat as a field reference. At the health zone level, Ebola response leaders responded to community feedback by improving public communications about the state of the epidemic and including in the updates a section on community feedback and answers to common questions.^21^ This information was shared both in print and on the radio. The leaders also made a range of modifications to Ebola response strategies to address community concerns, including hiring more local health care staff, involving Ebola survivors and traditional health care providers in response activities, and decreasing Ebola response visibility by reducing the number of vehicles used.^21^ In addition, vaccine eligibility was expanded in June 2019 to include a wider group of possible contacts,^27^ which resulted in a dramatic increase in vaccinations in the Beni health zone during the summer months. A compilation of available field reports documented more than 25 different response actions to which community feedback contributed.^21^ By mid-2019, in recognition of the benefits of community feedback in community engagement, the DRC Ministry of Health and WHO had incorporated a continuous community feedback collection objective into its strategic response planning document.^28^

At multiple levels, community feedback led to relatively rapid changes in practice.

## DISCUSSION

While other social science data collections took place during this Ebola outbreak,^2^^,^^21^^,^^29^^–^^31^ the Red Cross community feedback collection was unique in that it empowered local Red Cross volunteers to use the information immediately to address community questions and concerns, it was collected continuously throughout the outbreak, and it provided an opportunity for community members to talk about whatever concerned them in their own words. These attributes all took on additional importance, given the context of violence and insecurity of this outbreak.

Overall, the community feedback pointed to a need for more widespread and frequently updated risk communication and community engagement about cases, deaths, and survivors, as well as EVD symptoms. The lack of understanding that EVD often occurs without hemorrhagic symptoms seems to have contributed to the belief that people were being misdiagnosed with EVD, either unintentionally or intentionally. Similarly, comments about vaccination suggest that the principle of ring vaccination was not understood and that selectively vaccinating Ebola contacts caused suspicions of favoritism. These themes are supported by findings from other social science and news reports during the period.^2^^,^^21^^,^^29^^–^^33^

The community feedback pointed to a need for more widespread and frequently updated risk communication and community engagement.

The exploratory time-based analysis (Figure 2) shows that feedback about response activities (as compared with comments about Ebola itself or other topics indirectly related to response strategies) increased as the epidemic worsened, presumably because response activities in communities increased. Then, as case counts dropped, feedback about response activities also fell. However, statements about the mistreatment of bodies during safe and dignified burial and suggestions to expand ring vaccination to more people did not rise along with increasing cases in Beni. This pattern suggests that offering transparent body bags, widening vaccine eligibility. and other steps taken by the response alleviated these specific concerns. Comments about mistrust of Ebola treatment centers did not show a clear pattern overall, although it is notable that in Katwa and Butembo health zones in April and May 2019, a sudden increase in reported EVD cases was accompanied by an increase in the frequency of reported comments about distrust of Ebola treatment centers. Violent attacks on responders also occurred within roughly the same period.

The themes of government mistrust affecting willingness to participate in government-recommended outbreak control efforts are being seen in many communities during the COVID-19 pandemic.^34^^–^^36^ As with Ebola, mistrust of government efforts to control the spread of coronavirus affects people's willingness to perform behaviors that would protect themselves and reduce its spread.^37^ In addition, there are accusations that the response is politically driven or it is a way to make money, beliefs that coronavirus is not real, and frustrations with coronavirus disease diagnosis and treatment.

Throughout the outbreak, the utility of the Red Cross community feedback and all forms of social science inquiry were increasingly recognized as important to ending the epidemic. Not only was community feedback mentioned by WHO as part of its strategic plan, but several lessons learned documents from response leaders were published that emphasized the central role that community engagement and social science inquiry must play in emergency response, and presented examples of how it can be done.^5^^,^^38^^,^^39^ As a result of this work, IFRC has published a “Feedback starter kit” to guide volunteer groups in developing this capacity.^40^ IFRC and CDC also adapted the Ebola text-coding scheme to capture COVID-19-related feedback, and this framework is being used in several countries.^41^

### Limitations

Although a community feedback collection system was useful for hearing and addressing local concerns, important limitations were also present. Because Red Cross volunteers collected comments as they worked, comments were not systematically sampled, nor were they linked to any individual or household. Thus, estimating the prevalence of any given theme within the population was not possible, although it was possible to determine which sentiments were expressed more than others. Text coding is somewhat subjective, and coding text that has been paraphrased and then translated, sometimes twice before review, likely resulted in a loss of precision. When compared side-by-side with survey data from the same areas, community feedback has been found to emphasize negative feedback more than survey data. Therefore, such data are best viewed as qualitative, with only very limited quantification, and are ideally used in combination with data collected through other means.

## CONCLUSION

Despite these limitations, the Red Cross community feedback system filled an important gap during the Ebola outbreak in eastern DRC: the need for highly local, timely, open-ended, and continuous candid feedback from Ebola-affected communities. This information was used by Red Cross to adapt safe and dignified burial strategies to be more responsive to the community and supported changes in vaccine eligibility during the response. In any epidemic situation, as control strategies such as isolation, personal protection, contact tracing, and vaccination are introduced, communities can play a vital role in ensuring that strategies are implemented appropriately to receive maximum participation and effectiveness. Since every community is different, public health professionals need a way to monitor the reactions of communities as they introduce infection control measures. Community feedback can provide a tool for those willing to listen and act based on what they hear. This approach could not only result in more effective epidemic responses but also develop local community ownership of public health action and greater resilience in the face of any health threat.

## Supplementary Material

21-00144-Richardson-Supplement.docx

## Footnotes

* Safe and dignified burials are burials performed by specially trained Ebola response teams, in which participants are protected from the still-infectious body, but also can follow traditional practices to the extent possible.

† Volunteers were also trained to report any comments that related to threats against Red Cross or others immediately to their team leads, and the leads would report this information to the Red Cross safety and security team. IFRC's community engagement approach, grounded on listening and responding to questions and concerns and co-designing approaches with communities, has resulted in notably high levels of trust (including for performing Safe and Dignified burial interventions) and few instances of refusals or violence against volunteers, and has enabled the Red Cross to enter communities and neighborhoods that have traditionally been closed off to other service organizations.

## Translation

**En Français**

**Nouvelle approche à méthodes mixtes pour le suivi des perceptions communautaires d'Ebola et des efforts de réponse en République démocratique du Congo**

**Message clé:** Le système de feedback communautaire de la Croix-Rouge a permis la collecte et l'analyse rapides de plusieurs rapports verbaux pendant une épidémie d'Ebola dans l'est de la RDC. Grâce à ces informations, les responsables de la réponse à Ebola ont adapté des stratégies pour répondre aux préoccupations de la communauté. Lors d'une épidémie, le retour d'information de la communauté est essentiel pour s'assurer que les stratégies de réponse sont acceptées et appropriées.

**RÉSUMÉ**

**Contexte:** Les efforts visant à contenir la propagation d'Ebola dans l'est de la République démocratique du Congo (RDC) pendant l'épidémie 2018–2020 ont été confrontés à des défis pour gagner la confiance et la participation de la communauté. Cela a affecté la mise en œuvre des alertes communautaires, l'isolement précoce, la recherche des contacts, la vaccination ainsi que l'exercice des enterrements dignes et sécurisés. Pour comprendre rapidement les perspectives de la communauté et améliorer son engagement, des collaborateurs de la Croix-Rouge de la RDC, de la Fédération internationale de la Croix-Rouge et des Centres américains de contrôle et de prévention des maladies ont exploré une nouvelle méthode de collecte, de codage et d'analyse rapide des commentaires de la communauté.

**Méthodes:** Plus de 800 volontaires locaux de la Croix-Rouge de la RDC ont enregistré les questions et les commentaires non structurés et en texte libre des membres de la communauté pendant les activités de sensibilisation à Ebola. Les commentaires ont été codés et analysés en utilisant un système de codage de texte développé par les collaborateurs. Les commentaires codés ont ensuite été agrégés et regroupés qualitativement selon des thèmes principaux. Les tendances du moment ont également été examinées.

**Résultats:** Les communautés ont signalé un manque d'informations sur l'épidémie et la réponse, ainsi que des inquiétudes concernant le programme de vaccination contre Ebola et la qualité des soins de santé. Certains doutaient de la réalité d'Ebola. La réponse s'est servie des différentes réponses reçues pour réviser certaines approches d'engagement communautaire. Par exemple, deux changements de procédure ont été suivis d'une diminution des réponses négatives de la communauté: d'une part, l'utilisation de sacs mortuaires transparents (pour apaiser les craintes de vol de corps ou d'organes) et d'autre part, l'élargissement des critères d'éligibilité pour la vaccination contre le virus Ebola (pour répondre aux inquiétudes concernant l'injustice de la vaccination sélective des personnes au sein des communautés touchées par le virus).

**Discussion:** Ce système est unique en ce sens que les commentaires non structurés recueillis par les volontaires locaux dans le cadre de leur travail ont été rapidement codés, analysés et transmis aux autorités sanitaires afin qu'elles puissent les utiliser pour rectifier leur tir tout au long de la réponse. Il offre une plateforme permettant aux voix de la communauté de se faire entendre tout au long d'une intervention d'urgence. Également, ce système fournit un mécanisme pour évaluer les effets des ajustements du programme sur les sentiments de la communauté.

**Peer Reviewed**
