# Supplementary material for: New Mixed Methods Approach for Monitoring Community Perceptions of Ebola and Response Efforts in the Democratic Republic of the Congo
Source: Glob Health Sci Pract. 2021 Jun 30;9(2):332–43. doi: 10.9745/GHSP-D-21-00144 (PMC8324202; doi:10.9745/GHSP-D-21-00144)
Supplement: 21-00144-Richardson-Supplement.docx [file 21-00144-Richardson-Supplement.docx]

| **Supplement: Community feedback codes organized by theme*** | |
| --- | --- |
| **Theme** | **Code type** |
| **Ebola epidemic, reality of Ebola** | |
| Questions about the Ebola epidemic | Question |
| Perceptions, beliefs about Ebola outbreak locally | Statement (rumor, belief, observation) |
| Ebola does not exist | Statement (rumor, belief, observation) |
| End the Ebola outbreak | Suggestion/ request |
| Supernatural cause of EVD | Statement (rumor, belief, observation) |
| Duration of outbreak | Question |
| Ebola is real | Statement (rumor, belief, observation) |
| Exaggerated threat of Ebola | Statement (rumor, belief, observation) |
| Questions about EVD survivors | Question |
| Outcomes of Ebola outbreak | Statement (rumor, belief, observation) |
| Survivors of EVD | Statement (rumor, belief, observation) |
| Natural cause of EVD | Statement (rumor, belief, observation) |
| Denial of EVD outbreak | Other: refused dialogue^†^ |
| **Medical diagnosis and treatment** |  |
| Questions about EVD diagnosis, treatment | Question |
| Improve health care | Suggestion/ request |
| Mistrust of ETC (including allegedly harming people) | Statement (rumor, belief, observation) |
| Mistrust of medical care (incl. allegedly harming people) | Statement (rumor, belief, observation) |
| Questions about poor health care services | Question |
| EVD versus other diseases (includes misdiagnosing EVD) | Question |
| Provide medicines | Suggestion/ request |
| Thanks for the health care | Appreciation |
| Ebola has no cure, always results in death | Statement (rumor, belief, observation) |
| Provide testing for symptomatic people | Suggestion/ request |
| Traditional healers, private clinics caring for Ebola | Statement (rumor, belief, observation) |
| Follow up of EVD survivors | Suggestion/ request |
| **EVD vaccine and vaccination** |  |
| Expand or modify vaccination program | Suggestion/ request |
| Vaccine suspicions | Statement (rumor, belief, observation) |
| Questions about unfair vaccine distribution | Question |
| Other vaccine questions | Question |
| Vaccine effectiveness | Question |
| Vaccine safety | Question |
| Unfair vaccine distribution | Statement (rumor, belief, observation) |
| Vaccine non acceptance | Statement (rumor, belief, observation) |
| Vaccinate those at high risk | Suggestion/ request |
| **Ebola response feedback** |  |
| Questions about response processes | Question |
| Thank you, not further specified | Appreciation |
| Statements about response staff | Statement (rumor, belief, observation) |
| Expressions of encouragement | Appreciation |
| Call for flexible efficient action of response personnel | Suggestion/ request |
| Lack of trust in the response | Other: refused dialogue |
| Need for responsive government against Ebola | Suggestion/ request |
| Questions about action or inaction of government or other organizations | Question |
| Need for other materials or services to help in fighting Ebola | Suggestion/ request |
| Action or inaction government or others | Statement (rumor, belief, observation) |
| Mistrust in response materials or tools | Statement (rumor, belief, observation) |
| Disrespecting mores, customs | Statement (rumor, belief, observation) |
| **Ebola profit and politics** |  |
| Ebola is a “business” (or someone profiting) | Statement (rumor, belief, observation) |
| Ebola is a political tool | Statement (rumor, belief, observation) |
| Ebola is used for harming people (by political leaders or through political action) | Statement (rumor, belief, observation) |
| Ebola is a scheme of foreigners or others | Statement (rumor, belief, observation) |
| Financial motivations of response or responders | Other: refused dialogue |
| Response is a scheme of government or others | Other: refused dialogue |
| **Community engagement and awareness theme** |  |
| Thank you for Ebola awareness efforts | Appreciation |
| Community health promotion | Suggestion/ request |
| Train or involve certain people more | Suggestion/ request |
| Poor communication/ information | Statement (rumor, belief, observation) |
| Distribute educational materials | Suggestion/ request |
| Comment about Red Cross (agency or staff) | Statement (rumor, belief, observation) |
| **Washing and other infection control** |  |
| Provide handwashing station (with water, soap) | Suggestion/ request |
| Provide disinfectant products | Suggestion/ request |
| Pay for burned goods | Suggestion/ request |
| Provide protective clothing | Suggestion/ request |
| Destroy homes/latrines | Suggestion/ request |
| **Ebola disease and transmission theme** |  |
| EVD protective behaviors | Question |
| EVD transmission | Question |
| EVD facts and features | Question |
| EVD symptoms | Question |
| EVD origin | Question |
| Beliefs about EVD facts and features | Statement (rumor, belief, observation) |
| Animal related question | Question |
| How to take care of a sick person outside of a healthcare setting | Question |
| Sexual transmission prevention | Suggestion/ request |
| EVD prevention at school | Question |
| People spreading Ebola | Statement (rumor, belief, observation) |
| Protection of children, schools from Ebola | Statement (rumor, belief, observation) |
| Examine animals | Suggestion/ request |
| **Safe and dignified burial theme** |  |
| Burials | Question |
| Mutilation, theft of organs or cadavers | Statement (rumor, belief, observation) |
| Other suggestion, burials | Suggestion/ request |
| Other problems with SDB | Statement (rumor, belief, observation) |
| Lack of trust SDB | Statement (rumor, belief, observation) |
| Thanks to the SDB teams | Appreciation |
| Suggest increased family participation | Suggestion/ request |
| Suggest testing before burial | Suggestion/ request |
| Suggest taking photo of deceased | Suggestion/ request |
| Suggest building morgues | Suggestion/ request |
| **Points of entry, points of control theme** |  |
| Question about points of control (EVD checkpoints) | Question |
| Suggest blocking roads | Suggestion/ request |
| Suggestions related to points of control (EVD checkpoints) | Suggestion/ request |
| Travel, transport related questions | Suggestion/ request |
| Statements related to points of control (EVD checkpoints) | Statement (rumor, belief, observation) |
| Transportation-related issues | Question |
| **Other diseases and problems theme** |  |
| Need for social services outside the Ebola response | Suggestion/ request |
| Questions about the priority of EVD versus other problems | Question |
| **Security related theme** |  |
| Safety, security, end the war | Suggestion/ request |
| Description of a heard threat or observed violence | Other: sensitive or violence related^†^ |
| Mention of sexual exploitation or abuse | Other: sensitive or violence related |
| Refused dialogue due to security concerns | Other: refused dialogue |
| **Contact tracing theme** |  |
| Suggestions related to contact tracing | Suggestion/ request |
| Questions about contact tracing | Question |
| Statements about contact tracing | Statement (rumor, belief, observation) |
| **All other text codes, no theme** |  |
| Other suggestions | Suggestion/ request |
| Questions indicating suspicion | Question |
| Other rumor, belief, observation | Statement (rumor, belief, observation) |
| Other questions | Question |
| Prayers | Suggestion/ request |
| Other refusal | Other: refused dialogue |
| Specific case or incident | Question |
| Chased volunteers away | Other: refused dialogue |
| *Themes are ordered by frequency, with themes containing the most comments first. Within each theme, the comment text codes are ordered by frequency, with text codes containing the most comments first. The five most common themes are show in table 1. This list includes all codes and themes, with the most frequent at the top of the list and the least frequent at the bottom of the list.  ^†^ While these types were initially coded as “other” by volunteers, they were coded more specifically within “other” to denote people having refused to discuss Ebola with volunteers (refused dialogue) and those making statements about violence or threats of violence (sensitive or violence related), or statements about sexual exploitation (sensitive or violence related). | |
